# Impact of Tele-Intensive Care Units on the Clinical Outcomes of Critically Ill Patients With COVID-19: Retrospective Cohort Study

**DOI:** 10.2196/64996

**Published:** 2026-03-19

**Authors:** Thais Dias Midega, Fabio Barlem Hohmann, Renato Carneiro de Freitas Chaves, Guacyra Margarita Batista de Almeida, Vivian Jaqueline Lima Leoneza, Jennifer Ferreira Figueiredo Cabral, Bianca Veloso Vitalino, Emanuelle de Araújo Camboim, Nelma de Jesus Nogueira Machado, Ricardo Fernando Batista de Melo, Jorge Patrick Oliveira Feliciano, Breno Mendes Cardoso, Leonardo José Rolim Ferraz, Thiago Domingos Corrêa, Maura Cristina dos Santos, Renata Albaladejo Morbeck, Adriano José Pereira

**Affiliations:** 1 Department of Critical Care Medicine Hospital Israelita Albert Einstein São Paulo, Sao Paulo Brazil; 2 Massachusetts Institute of Technology Cambridge, MA United States; 3 Department of Critical Care Medicine Hospital de Emergência Dr. Daniel Houly Arapiraca Brazil; 4 Department of Critical Care Medicine Santa Casa de Misericórdia de Sorocaba Sorocaba Brazil; 5 Department of Critical Care Medicine Hospital Regional do Cariri Juazeiro do Norte Brazil; 6 Department of Critical Care Medicine Santa Casa de Misericórdia de São João del-Rei São João del-Rei Brazil; 7 Department of Critical Care Medicine Hospital Metropolitano Sul Dom Helder Câmara Cabo de Santo Agostinho Brazil; 8 Department of Critical Care Medicine Santa Casa de Misericórdia do Pará Belém Brazil; 9 Department of Critical Care Medicine Hospital Santa Lúcia Poços de Caldas Brazil; 10 Department of Critical Care Medicine Dom Orione Hospital Araguaína Brazil; 11 Department of Critical Care Medicine Hospital Municipal Senhora Santana Brasília de Minas Brazil; 12 Department of Telemedicine, Hospital Israelita Albert Einstein. São Paulo Brazil

**Keywords:** coronavirus, COVID-19, SARS-CoV-2, intensive care unit, telemedicine, tele–intensive care unit, Tele-ICU, clinical outcomes, mortality

## Abstract

**Background:**

The COVID-19 pandemic imposed an unprecedented demand for intensive care unit (ICU) resources in Brazil, where shortages of trained intensivists prompted the implementation of telemedicine-based critical care support strategies.

**Objective:**

This study aimed to evaluate the association between adherence to the Tele-ICU COVID-19 Brazil Program and clinical outcomes of ICU patients with COVID-19.

**Methods:**

We conducted a retrospective cohort study including all ICUs participating in the Tele-ICU COVID-19 Brazil Program between April and December 2020. Program adherence was assessed at 2 levels: patient coverage, defined as the number of daily multidisciplinary rounds per patient divided by the patient’s total ICU length of stay (LOS), and ICU coverage, defined as the number of daily multidisciplinary round days in the ICU divided by the total number of patient-days in that ICU. We compared outcomes between groups categorized by an empirically defined 50% cutoff: low patient coverage (<50%) versus high patient coverage (≥50%) and low ICU coverage (<50%) versus high ICU coverage (≥50%). Multilevel mixed-effects models accounting for ICU-level clustering were used to assess outcomes: logistic regression for ICU mortality (adjusted odds ratios) and linear mixed-effects regression with log-transformed ICU LOS (exponentiated coefficients, exp[β]).

**Results:**

A total of 1680 patients were included. Compared with the low patient coverage group (<50%), patients in the high patient coverage (≥50%) had lower Sequential Organ Failure Assessment scores (median 2, IQR 0-5 vs median 3, IQR 0-6; *P*=.007); shorter ICU LOS (median 6, IQR 3-11 days vs median 11, IQR 6-20 days; *P*<.001); and shorter hospital LOS (median 9, IQR 5-16 days vs median 14, IQR 8-26 days; *P*<.001). In unadjusted analyses, ICU mortality did not differ significantly between the low and high patient coverage groups (50.1% vs 46.3%; *P*=.16). In multilevel analysis, mechanical ventilation and vasopressor use were independently associated with higher ICU mortality. Higher patient coverage was independently associated with lower ICU mortality (adjusted odds ratio 0.52, 95% CI 0.27-0.99; *P*=.048). In the log-transformed mixed-effects model for ICU LOS, a higher Sequential Organ Failure Assessment score (exp[β] 1.037, 95% CI 1.02-1.05; *P*<.001) and use of mechanical ventilation (exp[β] 1.23, 95% CI 1.05-1.43; *P*=.01) were associated with longer ICU LOS, whereas higher patient coverage was independently associated with shorter ICU LOS (exp[β] 0.17, 95% CI 0.13-0.21; *P*<.001). ICU coverage was not independently associated with ICU mortality or ICU LOS.

**Conclusions:**

Greater patient-level coverage by remote intensivist–led multidisciplinary rounds within the Tele-ICU program was independently associated with lower ICU mortality and shorter ICU LOS. These findings support the potential contribution of tele–critical care strategies to expanding specialist support during public health emergencies.

## Introduction

### Background

COVID-19 has become a major public health care concern, with almost half a billion cases diagnosed and more than 6 million deaths reported across the globe [1]. During the pandemic, waves of increased number of newly diagnosed patients with SARS-CoV-2 infection were reported worldwide, with varied degrees of disease severity [2]. The rapid spread of SARS-CoV-2 increased the demand for intensive care units (ICUs) and the pressure on health care systems [2].

In Brazil, the high demand for ICU beds coupled with the shortage of intensivists to care for patients who are critically ill—particularly in rural and remote regions far from major urban centers—motivated the search for alternative strategies to deliver efficient care [3]. In this scenario, in a partnership between the Brazilian Ministry of Health (via the Institutional Development Support Program of the Unified Health System) and some of the top-ranked Brazilian hospitals, the Tele-ICU COVID-19 Brazil Program was created to provide remote intensivists to guide daily multidisciplinary rounds (DMRs) in public COVID-19 ICUs from Brazil with patients with COVID-19. The program consisted of DMRs (structured patient-centered discussions) with the remote intensivist and the local ICU multidisciplinary team, aiming to review diagnostic hypotheses, list active problems, and establish joint therapeutic goals.

Different Tele-ICU models exist and vary in structure and intensity. The most common is the centralized “hub-and-spoke” model, where a central team remotely monitors several ICUs [4]. Alternatively, decentralized models allow remote specialists to provide support from multiple locations on demand. These configurations may include continuous monitoring, scheduled virtual rounds, or reactive consultations, depending on institutional needs and available infrastructure [4].

Observational studies indicated that implementing Tele-ICU programs may be an effective strategy for improving clinical outcomes [5-11]. However, reported impacts vary across studies, likely due to differences in Tele-ICU models and applications, including second opinion consultations, real-time evaluations, remotely conducted rounds, and others [12-14]. More recently, a randomized clinical trial evaluating an intensivist-led, telemedicine-based strategy that incorporated DMRs found no significant improvement in ICU length of stay (LOS) or patient mortality [15].

However, evidence regarding the impact of Tele-ICU interventions on clinical outcomes, particularly during large-scale health care crises such as the COVID-19 pandemic, remains limited [7,8]. In this context, this study aimed to evaluate the association of the Tele-ICU COVID-19 Brazil Program and clinical outcomes of patients with COVID-19. We hypothesized that higher adherence to the program’s structured DMRs would be associated with lower ICU mortality and shorter ICU LOS among patients admitted to public ICUs across Brazil during the COVID-19 pandemic.

### Objectives

This study aimed to evaluate the association of the adherence to the Tele-ICU COVID-19 Brazil Program and clinical outcomes of patients with COVID-19.

## Methods

### Study Design

We performed a retrospective study to evaluate the impact of Tele-ICU COVID-19 Brazil Program on clinical outcomes of ICU patients with COVID-19.

### Ethical Considerations

The study was approved by Brazilian National Ethics Committee on August 27, 2020, with waiver of informed consent (CAAE: 31459120.0.0000.0071, opinion number: 4.240.138). All data used in the study were deidentified before analysis to protect participant privacy and ensure confidentiality. No compensation or reimbursement was provided to participants.

### Tele-ICU Program

The Tele-ICU COVID-19 Brazil Program was led by the Tele-ICU Department of the Hospital Israelita Albert Einstein, a private quaternary care hospital located in São Paulo, Brazil. Participating ICUs that accepted the invitation (a convenience sample) were located in public hospitals across different geographic regions of the country (1 ICU per hospital) and were designated by the Brazilian Ministry of Health between April and December 2020. The recruitment process occurred at the institutional level and was coordinated directly by the Ministry of Health, which was responsible for identifying, evaluating, and selecting eligible ICUs. ICUs were eligible if they belonged to public or philanthropic hospitals and did not have a board-certified intensivist providing daily on-site care. The Ministry also established criteria for replacing units during the program: ICUs could be removed if they missed daily tele-rounds for 5 consecutive days or for more than 10 nonconsecutive days within a month.

As critical care specialists were not available daily in the participating ICUs during this period, DMRs were conducted entirely by the program’s remote intensivists. Discussions were conducted by a board-certified intensivist trained in telemedicine, based at the remote center (tele-intensivist, in São Paulo, Brazil), in collaboration with the local multidisciplinary team (physician, nurse, and physiotherapist, in any part of the country). DMRs took place from Monday to Friday at a predetermined time (mostly during the mornings), from April to December 2020, and approached all patients admitted to the participating ICUs. The main objective of the DMR conducted by the tele-intensivist was to review diagnostic hypotheses, list active problems, and jointly create a treatment plan until the next DMR. Tele-intensivists made recommendations based on updated scientific evidence, suitable to the local context. Clinical protocols in texts and figures were made accessible through a dedicated application developed for the physicians and multidisciplinary team of the participating ICUs.

### Study Participants

All the 17 participant ICUs of the program were invited to participate in this study. All patients admitted to these ICUs during the Tele-ICU program, from April to December 2020, were included in this analysis.

We excluded patients who meet the following criteria: (1) aged <18 years, (2) receiving exclusive palliative care at ICU admission, and (3) incomplete data about ICU outcomes.

### Data Collection and Study Variables

All study data were collected by the Tele-ICU intensivist during the DMRs performed during the program and complemented by the local staff of the participant ICUs. Data were stored on a server at Hospital Israelita Albert Einstein, São Paulo, Brazil, and retrieved by the study authors after the approval of the ethics committee.

Collected data included demographics, comorbidities, Sequential Organ Failure Assessment (SOFA) score [16], number of DMRs, resource use, and organ support (vasopressors, noninvasive ventilation, and mechanical ventilation) during ICU stay, palliative care, ICU and hospital LOS, and ICU and in-hospital mortality. Variables were collected during the DMRs (and lately complemented by invited professionals of the participant ICU). SOFA score refers to the maximum value of SOFA during ICU stay.

### Definitions

To evaluate the impact of the Tele-ICU program, we developed 2 variables: patient coverage and ICU coverage. *Patient coverage* quantifies how often each patient’s case was discussed during DMRs based on the hypothesis that more frequent discussions could enhance clinical decision-making and continuity of care. *ICU coverage* reflects the overall participation of each ICU in the program, hypothesizing that higher engagement would promote local learning and greater adherence to evidence-based practices through regular interaction with remote intensivists. These variables were defined as follows:

- Patient coverage=(number of DMRs per patient)/(patient’s ICU LOS)
- ICU coverage=(number of DMR days in the ICU)/(total ICU patient-days)

A cutoff of 50% was empirically defined to categorize groups. Patients were classified into *low patient coverage* (<50%) and *high patient coverage* (≥50%) groups based on their individual patient coverage. Similarly, ICUs were categorized into low ICU coverage (<50%) and high ICU coverage (≥50%) groups based on their overall ICU coverage.

### Outcomes

Our primary outcome of interest was ICU mortality. The secondary outcomes were ICU LOS and use of resources (eg, mechanical ventilation, noninvasive ventilation, use of vasopressors, and renal replacement therapy).

### Statistical Analysis

Categorical variables are presented as absolute and relative frequencies. Continuous variables are presented as median with IQR. Normality was assessed using the Shapiro-Wilk test.

Comparisons were made between groups: low patient coverage group vs high patient coverage group and low ICU coverage group vs high ICU coverage group. Continuous variables were compared using the independent 2-tailed *t* test or the Mann-Whitney *U* test in case of nonnormal distribution. Univariate analyses were performed to identify which predictors were associated with ICU mortality and ICU LOS.

We performed multivariate analyses using multilevel mixed modeling, with hospitals as random effect. All variables tested with a *P* value <.20 in the univariate analyses were included as fixed effects. We performed a multicollinearity analysis before the backward elimination procedure. A mixed model was undertaken to obtain adjusted odds ratio (OR) along with 95% CI and to define which variables were independently associated with ICU mortality.

A linear mixed-effects model with log-transformed ICU LOS as the outcome was used, with hospital as a random effect. Exp(β) along with the 95% CI were obtained to define which variables were independently associated with ICU LOS. To perform the analysis of the predictor ICU LOS, probability distribution analyses of the outcome were conducted. We carried out inferential tests to assess the fit of various probability distributions. The only distribution that demonstrated adherence was the normal distribution for data transformed by natural logarithm. Thus, the outcome variable ICU LOS was transformed by taking the natural logarithm (ln).

All statistical tests were 2-tailed, and *P*<.05 was considered statistically significant. Analyses were performed using R software (version 4.1.0; R Foundation for Statistical Computing).

## Results

From April to December 2020, a total of 1945 patients from 17 different ICUs across different Brazilian geographic regions were included in the database and had their plan of care defined by the remote tele-intensivist on at least 1 day of the Tele-ICU program. We excluded 233 (11.9%) patients who lacked data on ICU outcomes (leading to the exclusion of an entire ICU, as none of the patients from this ICU had available outcome data); 18 (0.9%) patients aged younger than 18 years; and 14 (0.7%) patients receiving palliative care since the ICU admission, as previously defined in our study protocol. Finally, 1680 patients from 16 different ICUs were included in the study.

Baseline characteristics of patients included in the study are presented in Table 1. The median age in this cohort was 66 (IQR 55-76) years, and 56.4% (865/1534) of the patients were men. The median SOFA score was 4 (IQR 1-9), and the median number of DMRs per patient was 3 (IQR 2-6). In this cohort, during ICU stay, 56.8% (769/1354) of the patients required mechanical ventilation, 46.3% (627/1354) of the patients required vasopressors, 16.9% (229/1354) of the patients required renal replacement therapy, and 9.7% (132/1361) of the patients required noninvasive ventilation. ICU mortality was 48.3% (812/1680), and the hospital mortality was 51.1% (847/1658). The median ICU and hospital LOS were, respectively, 8 (IQR 4-15) days and 11 (IQR 6-20) days (Table 1).

**Table 1:** Baseline characteristics and clinical outcomes of intensive care unit (ICU) patients with COVID-19 admitted to public ICUs participating in the Tele-ICU COVID-19 Brazil Program (retrospective multicenter cohort study, Brazil, April to December 2020; N=1680)a.

Characteristics			Patients
Male, n (%)			865 (56.4)
Age (years), median (IQR)			66 (55-76)
**Comorbidities, n (%)**			
	Systemic hypertension	684 (62.8)	
	Diabetes mellitus	441 (40.5)	
	Congestive heart failure	74 (6.8)	
	Acute cerebral stroke	60 (5.5)	
	Coronary arterial disease	17 (1.6)	
	Previous myocardial infarction	21 (1.9)	
	Asthma	27 (2.5)	
	COPD^b^	86 (7.9)	
	Chronic kidney disease	44 (4)	
	Chronic kidney disease requiring RRT^c^	19 (1.7)	
	Locoregional cancer	4 (0.4)	
	Metastatic cancer	5 (0.5)	
	Hematologic cancer	1 (0.1)	
	Cognitive impairment and dementia	19 (1.7)	
	Liver cirrhosis	1 (0.1)	
ICU mortality, n (%)			812 (48.3)
Hospital mortality, n (%)			847 (51.1)
ICU LOS^d^, median (IQR)			8 (4-15)
Hospital LOS, median (IQR)			11 (6-20)
SOFA^e^ score, median (IQR)			4 (1-9)
Number of DMRs^f^ per patient, median (IQR)			3 (2-6)
**Support on the first day of the Tele-ICU visit, n (%)**			
	Mechanical ventilation	639 (47.2)	
	Noninvasive ventilation	132 (9.7)	
	Vasopressors	425 (31.4)	
	RRT	128 (9.5)	
**Support in the first 24 hours of ICU stay, n (%)**			
	Mechanical ventilation	243 (36.9)	
	Noninvasive ventilation	71 (10.8)	
	Vasopressors	164 (24.9)	
	RRT	26 (3.9)	
**Support at any time during ICU stay, n (%)**			
	Mechanical ventilation	769 (56.8)	
	Noninvasive ventilation	168 (12.4)	
	Vasopressors	627 (46.3)	
	RRT	229 (16.9)	

^a^Percentages were calculated based on available data for each variable.

^b^COPD: chronic obstructive pulmonary disease.

^c^RRT: renal replacement therapy.

^d^LOS: length of stay.

^e^SOFA score: Sequential Organ Failure Assessment score, ranges from 0 to 24, with higher scores indicating more severe organ dysfunction.

^f^DMR: daily multidisciplinary round.

Compared with the low patient coverage group, patients in the high patient coverage group had shorter ICU LOS (median 6, IQR 3-11 days vs median 11, IQR 6-20 days; *P*<.001) and hospital LOS (median 9, IQR 5-16 days vs median 14, IQR 8-26 days; *P*<.001; Table 2). SOFA score was higher in the low patient coverage group (median 3, IQR 0-6 vs median 2, IQR 0-5; *P*=.007). ICU mortality (50.1% vs 46.3%; *P*=.16) did not differ significantly between the low and high patient coverage groups. During ICU stay, patients in the high patient coverage group used mechanical ventilation less frequently, but used noninvasive ventilation more frequently when compared with the low patient coverage group.

**Table 2:** Comparison of clinical outcomes and resource use according to patient-level adherence to tele–intensive care unit (ICU) daily multidisciplinary rounds among ICU patients with COVID-19 (Brazil, April to December 2020).

		Low patient coverage group (n=673)	High patient coverage group (n=862)	*P* value
ICU deaths, n (%)		337 (50.1)	399 (46.3)	.16^a^
Hospital deaths, n (%)		351 (52.2)	417 (48.4)	.17^a^
ICU LOS^b^, median (IQR)		11 (6-20)	6 (3-11)	<.001^c^
Hospital LOS, median (IQR)		14 (8-26)	9 (5-16)	<.001^c^
SOFA^d^ score, median (IQR)		3 (0-6)	2 (0-5)	.007^c^
**Support in the first 24 hours of ICU stay, n (%)**				
	Mechanical ventilation	86 (45.7)	154 (33)	.003^a^
	Noninvasive ventilation	13 (6.9)	58 (12.4)	.06^a^
	Vasopressors	56 (29.8)	106 (22.7)	.07^a^
	RRT^e^	11 (5.9)	14 (3)	.14^a^
**Support at any time during ICU stay, n (%)**				
	Mechanical ventilation	328 (60.3)	357 (51.6)	.003^a^
	Noninvasive ventilation	58 (10.7)	108 (15.6)	.01^a^
	Vasopressors	261 (48)	301 (43.5)	.13^a^
	RRT	105 (19.3)	100 (14.5)	.03^a^

^a^*P* values were calculated using the chi-square test.

^b^LOS: length of stay.

^c^*P* values were calculated using the Mann-Whitney *U* test.

^d^SOFA score: Sequential Organ Failure Assessment score, ranges from 0 to 24, with higher scores indicating more severe organ dysfunction.

^e^RRT: renal replacement therapy.

Compared with the low ICU coverage group, patients in the high ICU coverage group had lower SOFA scores (median 2, IQR 0-4 vs median 3, IQR 1-7; *P*<.001), lower ICU mortality (44.3% vs 52.5%; *P*=.004), lower ICU LOS (median 7, IQR 4-12 days vs median 9, IQR 5-17 days; *P*<.001), and lower hospital LOS (median 8, IQR 5-16 days vs median 14, IQR 7-23 days; *P*<.001). During ICU stay, patients in the high ICU coverage group used mechanical ventilation, noninvasive ventilation, vasopressors, and renal replacement therapy less frequently (Table 3).

**Table 3:** Comparison of clinical outcomes and resource use according to intensive care unit (ICU)–level adherence to the Tele-ICU COVID-19 Brazil Program (Brazil, April to December 2020).

		Low ICU coverage group (n=844)	High ICU coverage group (n=836)	*P* value
ICU deaths, n (%)		442 (52.5)	370 (44.3)	.004^a^
Hospital deaths, n (%)		451 (53.5)	396 (48.7)	.05^a^
ICU LOS^b^, median (IQR)		9 (5-17)	7 (4-12)	<.001^c^
Hospital LOS, median (IQR)		14 (7-23)	8 (5-16)	<.001^c^
SOFA^d^ score, median (IQR)		3 (1-7)	2 (0-4)	<.001^c^
Palliative care, n (%)		146 (17.3)	123 (14.7)	.17^a^
**Support in the first 24 hours of ICU stay, n (%)**				
	Mechanical ventilation	142 (44)	101 (30.1)	<.001^a^
	Noninvasive ventilation	32 (9.9)	39 (11.6)	.56^a^
	Vasopressors	93 (28.8)	71 (21.1)	.03^a^
	RRT^e^	24 (7.4)	2 (0.6)	<.001^a^
**Support at any time during ICU stay, n (%)**				
	Mechanical ventilation	471 (63.1)	298 (49)	<.001^a^
	Noninvasive ventilation	109 (14.6)	59 (9.7)	.008^a^
	Vasopressors	384 (51.5)	243 (40)	<.001^a^
	RRT	168 (22.5)	61 (10)	<.001^a^

^a^*P* values were calculated using the chi-square test.

^b^LOS: length of stay.

^c^*P* values were calculated using the Mann-Whitney *U* test.

^d^SOFA score: Sequential Organ Failure Assessment score; ranges from 0 to 24, with higher scores indicating more severe organ dysfunction.

^e^RRT: renal replacement therapy.

Independent predictors of ICU mortality were the use of mechanical ventilation (OR 3.22, 95% CI 2.056-5.043; *P*<.001) and vasopressors (OR 1.87, 95% CI 1.236-2.824; *P*=.003) during ICU stay. The use of noninvasive ventilation (OR 0.54, 95% CI 0.331-0.881; *P*=.01) and patient coverage (OR 0.520, 95% CI 0.272-0.993; *P*=.048) were identified as a protective factors (Table 4). Neither ICU coverage nor total time of ICU participation in the project remained in the final model.

**Table 4:** Multilevel mixed-effects logistic regression analysis of factors associated with intensive care unit (ICU) mortality among critically ill patients with COVID-19 enrolled in the Tele-ICU COVID-19 Brazil Program (Brazil, April to December 2020).

Fixed effects	OR^a^ (95% CI)	*P* value
SOFA^b^ score	1.238 (0.860-1.317)	.052
Use of MV^c^ during ICU stay	3.222 (2.056-5.043)	<.001
Use of NIV^d^ during ICU stay	0.540 (0.331-0.881)	.01
Use of vasopressor during ICU stay	1.870 (1.236-2.824)	.003
Patient coverage	0.520 (0.272-0.993)	.048

^a^OR: odds ratio.

^b^SOFA: Sequential Organ Failure Assessment.

^c^MV: mechanical ventilation.

^d^NIV: noninvasive ventilation.

Independent predictors of higher ICU LOS included in the final adjusted model were SOFA (exp[β] 1.037, 95% CI 1.02-1.05; *P*<.001), use of mechanical ventilation during ICU stay (exp[β] 1.228, 95% CI 1.05-1.43; *P*=.01), and patient coverage as protective factor (exp[β] 0.166, 95% CI 0.13-0.21; *P*<.001; Table 5). As in the first model for ICU mortality, neither ICU coverage nor total time of ICU participation in the project remained in this final adjusted model.

**Table 5:** Multilevel linear mixed-effects model evaluating factors associated with intensive care unit (ICU) length of stay among critically ill patients with COVID-19 participating in the Tele-ICU COVID-19 Brazil Program (Brazil, April to December 2020).

Fixed effects	Exp(β)^a^ (95% CI)	*P* value
SOFA^b^ score	1.037 (1.02-1.05)	<.001
Use of MV^c^ during ICU stay	1.228 (1.05-1.43)	.01
Patient coverage	0.166 (0.13-0.21)	<.001

^a^Exp(β): exponentiated regression coefficient from the linear mixed-effects model with log-transformed ICU LOS; values >1 indicate longer ICU LOS, and values <1 indicate shorter ICU LOS.

^b^SOFA score: Sequential Organ Failure Assessment score; ranges from 0 to 24, with higher scores indicating more severe organ dysfunction.

^c^MV: mechanical ventilation.

## Discussion

### Principal Findings

The main finding of this study was that patients with higher patient coverage by Tele-ICU, defined as a greater proportion of ICU days during which patients were discussed in DMRs led by a remote intensivist during the Tele-ICU COVID-19 Brazil Program, were associated with lower ICU mortality and LOS. We postulate that Tele-ICU may have improved clinical outcomes by implementing multidisciplinary rounds, thereby enhancing adherence to best practices and promoting continuity of care in participating ICUs.

Our results are in line with some previous studies regarding the association between the use of Tele-ICU and lower mortality and LOS [5-10]. For instance, a prospective study found that the implementation of a Tele-ICU intervention, where the Tele-ICU team participated in critical care processes throughout the day, was associated with reduced adjusted odds of mortality and reduced hospital LOS, as well as with changes in best practice adherence and lower rates of preventable complications [17]. Similarly, a before-and-after study observed a notable rise in the percentage of patients receiving a daily sedative interruption with Tele-ICU support, which involved the incorporation of a third shift of Tele-ICU pharmacist assistance [18].

In addition, recently, a cluster randomized controlled trial found that telemedical quality improvement program increased adherence to 7 evidence-based German performance indicators in acute ICU care [19]. The quality indicators included were sedation, analgesia and delirium, ventilation, weaning from ventilation, infection management, enteral nutrition, patient and family communication, and early mobilization [19].

Nevertheless, there is significant heterogeneity among various Tele-ICU models, making these studies potentially noncomparable [8,9]. There are notable discrepancies concerning various characteristics in the previous studies regarding the technology and hospitals used, as well as the autonomy of Tele-ICU intensivists [8,9,18].

Tele-ICU COVID-19 Brazil Program was based on the implementation of multidisciplinary rounds conducted by a board-certified tele-intensivist in ICUs lacking daily critical care specialists. Daily rounds conducted by an intensivist have been previously associated with a 3-fold reduction in hospital mortality in surgical ICUs [20]. Furthermore, a study exploring organizational factors in Brazilian ICUs did not identify a direct impact of regular multidisciplinary rounds on mortality; however, the findings suggest that collaborative multidisciplinary efforts among ICU care providers positively influence patient outcomes [21].

The Tele-ICU program was also linked to an increase in the use of noninvasive ventilation and a decrease in the need of invasive mechanical ventilation. We hypothesize that the adoption of noninvasive ventilation increased as intensivists encouraged its use, and local teams observed that patients responding positively to noninvasive ventilation could safely be managed (especially considering the fear of aerosolization and spread of SARS-CoV-2 in the environment, which was highly prevalent in the first COVID-19 wave).

Interestingly, no independent association was found between overall ICU engagement (ICU coverage) or duration of ICU participation in the program and patient outcomes. Although we initially hypothesized that greater program exposure or broader ICU involvement would lead to better results, our findings suggest that the direct and consistent implementation of daily Tele-ICU rounds may have been the most influential factor, rather than general program duration or institutional engagement.

The program may also have enhanced continuity of care, as the remote intensivist overseeing the rounds remained mostly consistent from Monday to Friday, whereas the local team members often changed daily during the weekdays. Continuity of care is known to be a fundamental aspect of effective medical treatment and is linked to enhanced patient satisfaction, greater use of care services, and reduced health care costs [22]. Maintaining continuity of care in the ICU environment is particularly challenging, especially due to the prolonged duty hours that can impact the well-being of physicians [23]. In this scenario, the Tele-ICU solution emerges as a potential solution, providing a more stable and continuous presence to support ongoing patient care.

### Limitations

Finally, our study has some limitations. First, it has a retrospective design, thus reporting associations rather than cause-and-effect relationships. Second, we used the maximum SOFA score to quantify the severity of illness in patients, which may overestimate the severity of patients with COVID-19 included in our analysis. Third, although our study includes a considerable number of patients from multiple ICUs across different regions, the Tele-ICU program itself was coordinated by a single center, which may limit the external validity of the findings. Fourth, we acknowledge the potential variability in the presence of local intensivists across participating ICUs. While all selected ICUs lacked full-time intensivist coverage, which was a key inclusion criterion, there may have been occasional availability of specialists in some sites. This unmeasured variability may have influenced local care dynamics and should be considered as a potential confounding factor in interpreting the results. Finally, data on tracheostomy procedures were not collected in this study, which prevents evaluation of their potential impact on outcomes such as ICU mortality, duration of mechanical ventilation, and LOS [24]. Therefore, these should be considered when assessing generalizability, and the study should be considered as hypothesis generating.

### Conclusions

During the COVID-19 pandemic, the Tele-ICU COVID-19 Brazil Program showed that greater exposure to DMRs led by remote intensivists was associated with lower ICU mortality and shorter ICU stays. These findings suggest that structured tele–critical care support may help extend specialized expertise, promote adherence to best practices, and enhance continuity of care in settings with limited intensivist availability. The results also highlight the potential value of Tele-ICU models in low- and middle-income countries and during periods of health care system strain. Further research is needed to confirm these findings, identify which program components drive the greatest benefit, and evaluate different Tele-ICU configurations and their cost-effectiveness to support future implementation efforts.

## Abbreviations

DMR: daily multidisciplinary round
ICU: intensive care unit
LOS: length of stay
OR: odds ratio
SOFA: Sequential Organ Failure Assessment

## Data Availability

The datasets analyzed during this study are not publicly available due to ethical and regulatory restrictions, including requirements from Brazilian regulatory agencies. However, anonymized data may be made available from the corresponding author upon reasonable request, subject to appropriate regulatory approvals.
